# The Early Psychosis Intervention Center (EPICENTER): development and six-month outcomes of an American first-episode psychosis clinical service

**DOI:** 10.1186/s12888-015-0650-3

**Published:** 2015-10-28

**Authors:** Nicholas J. K. Breitborde, Emily K. Bell, David Dawley, Cindy Woolverton, Alan Ceaser, Allison C. Waters, Spencer C. Dawson, Andrew W. Bismark, Angelina J. Polsinelli, Lisa Bartolomeo, Jessica Simmons, Beth Bernstein, Patricia Harrison-Monroe

**Affiliations:** 1 Department of Psychiatry and Behavioral Health, The Ohio State University, Columbus, Ohio USA; 2 Department of Psychiatry, The University of Arizona, Tucson, Arizona USA; 3 Department of Psychology, The University of Arizona, Tucson, Arizona USA; 4 Department of Psychiatry and Behavioral Sciences, Stanford University, Stanford, California USA; 5 Department of Psychiatry and Behavioral Sciences, Emory University School of Medicine, Atlanta, Georgia USA; 6 VISN-22 Mental Illness, Research, Education and Clinical Center (MIRECC), VA San Diego Healthcare System, San Diego, California USA; 7 Department of Education, The University of Arizona, Tucson, Arizona USA

**Keywords:** First-episode psychosis, Treatment, Cognitive behavioral therapy, Family psychoeducation, Cognitive remediation, Cost-effectiveness

## Abstract

**Background:**

There is growing evidence that specialized clinical services targeted toward individuals early in the course of a psychotic illness may be effective in reducing both the clinical and economic burden associated with these illnesses. Unfortunately, the United States has lagged behind other countries in the delivery of specialized, multi-component care to individuals early in the course of a psychotic illness. A key factor contributing to this lag is the limited available data demonstrating the clinical benefits and cost-effectiveness of early intervention for psychosis among individuals served by the American mental health system. Thus, the goal of this study is to present clinical and cost outcome data with regard to a first-episode psychosis treatment center within the American mental health system: the Early Psychosis Intervention Center (EPICENTER).

**Methods:**

Sixty-eight consecutively enrolled individuals with first-episode psychosis completed assessments of symptomatology, social functioning, educational/vocational functioning, cognitive functioning, substance use, and service utilization upon enrollment in EPICENTER and after 6 months of EPICENTER care. All participants were provided with access to a multi-component treatment package comprised of cognitive behavioral therapy, family psychoeducation, and metacognitive remediation.

**Results:**

Over the first 6 months of EPICENTER care, participants experienced improvements in symptomatology, social functioning, educational/vocational functioning, cognitive functioning, and substance abuse. The average cost of care during the first 6 months of EPICENTER participation was lower than the average cost during the 6-months prior to joining EPICENTER. These savings occurred despite the additional costs associated with the receipt of EPICENTER care and were driven primarily by reductions in the utilization of inpatient psychiatric services and contacts with the legal system.

**Conclusions:**

The results of our study suggest that multi-component interventions for first-episode psychosis provided in the US mental health system may be both clinically-beneficial and cost-effective. Although additional research is needed, these findings provide preliminary support for the growing delivery of specialized multi-component interventions for first-episode psychosis within the United States.

**Trial registration:**

ClinicalTrials.gov Identifier: NCT01570972; Date of Trial Registration: November 7, 2011

## Background

Psychotic disorders exert a significant burden under current systems of care worldwide. Despite advances in the treatment of these disorders, individuals with psychosis typically experience a course of illness characterized by repeated relapses of psychotic symptoms [1], persistent unemployment [2], limited social relationships [3], and premature mortality [4]. Not surprisingly, the cost of care for psychotic disorders is astronomical. For example, within the United States, the cost of care in 2002 for a single psychotic disorder (i.e., schizophrenia) was nearly 63 billion dollars [5], suggesting that in 2015 the cost will exceed $81 billion dollars after adjusting for inflation [6].

There is growing evidence that specialized clinical services targeted toward individuals early in the course of a psychotic illness may be effective in reducing both the clinical and economic burden associated with these illnesses [7–11]. The success of these early intervention services has sparked significant health services reform worldwide, including the establishment of a national early intervention service network in the United Kingdom [12] and the funding of a similar national care system by the federal government of Australia [13].

Unfortunately, the United States has lagged behind other countries in the delivery of specialized, multi-component care to individuals early in the course of a psychotic illness [14]. A key factor contributing to this lag is the limited available data demonstrating the clinical benefits and cost-effectiveness of early intervention for psychosis among individuals served by the American mental health system ([14, 15], however, see [11, 16, 17]). Consequently, there is a clear need for additional systematic evaluations of multi-component treatment packages for individuals with first-episode psychosis in the United States.

Thus, the goal of this manuscript is twofold. First, we will review the process of developing a multi-component treatment program for individuals with first-episode psychosis within the American mental health system: the Early Psychosis Intervention Center (EPICENTER: [18, 19]). Established in 2010 within the Department of Psychiatry at the University of Arizona, this program serves a catchment area of approximately 1 million individuals in the southwest United States. Second, we will review clinical and cost data among EPICENTER participants during the 6 months prior to joining EPICENTER as compared to during the first 6 months of EPICENTER care. The purpose of this activity is to quantify the possible clinical and economic benefits associated with EPICENTER participation.

## Methods

Human subject research completed as part of this project was approved by the University of Arizona Institutional Review Board (Project Numbers 09-1113-02 and 10-0440-02) and was completed in compliance with the Helsinki Declaration.

### Participants

**Fig. 1:**
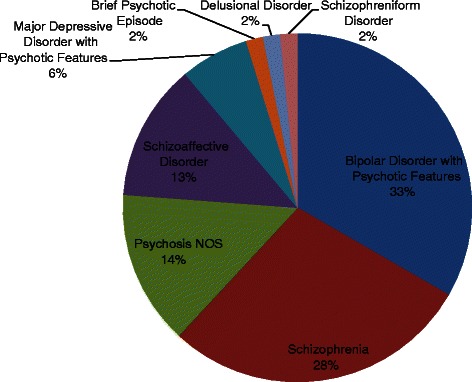
Distribution of Psychotic Disorder Diagnoses among EPICENTER Participants

Participants in this study were the first 68 individuals with first-episode psychosis consecutively enrolled at EPICENTER. Eligibility criteria for EPICENTER include: diagnosis of a schizophrenia-spectrum disorder or affective disorder with psychotic features as determined using the Structured Clinical Interview for the DSM-IV-TR [20]; onset of psychotic symptoms within the past 5 years per the Symptom Onset in Schizophrenia Inventory [21]; ages 15–35; and no evidence of mental retardation or organic brain impairment as evidenced by a premorbid IQ greater than 70 as estimated using the Reading subtest of the Wide Range Achievement Test [22]. Among the current sample, there were 49 men and 19 women with an average age of 22.71 years. The median duration of time since the onset of psychotic symptoms was 16.76 months. The distribution of psychotic disorder diagnoses is summarized in Fig. 1. Seventy-eight percent were prescribed antipsychotic medication prior to enrollment in EPICENTER, and none had previously participated in evidence-based psychosocial treatment for psychosis.

During the time in which these 68 individuals were consecutively enrolled in EPICENTER, a total of 264 individuals were referred to this clinical service. Forty-seven of these referred individuals were excluded from participation due to being younger than 15 years (*n* = 17), not having psychosis (*n* = 12), having a first onset of psychotic symptoms > 5 years prior (*n* = 10), being older than 35 years (*n* = 4), having an estimated premorbid IQ < 70 (*n* = 2), having psychosis resulting from a general medical condition (*n* = 1), and having attenuated psychotic symptoms that did not meet DSM-IV criteria for a psychotic disorder (*n* = 1). An additional 149 were referred to the study but declined to participate in the eligibility assessment.

Written informed consent with regard to study participation was obtained from all adult subjects. For participants under the age of 18, written informed consent was obtained from the participant’s parent or guardian and written assent was obtained from the participant.

### Procedures

Individuals with first-episode psychosis completed the measures described below as part of a larger research battery designed to investigate mediators and moderators of treatment response among individuals with first-episode psychosis [19, 23]. All measures were administered upon enrollment in EPICENTER and after 6 months of participation in EPICENTER services. When possible, assessments were administered by blinded researchers. However, in some instances, baseline assessments (i.e., assessments completed prior to the start of EPICENTER care) were completed by EPICENTER clinical staff due to staffing limitations.

### Measures

#### Symptomatology

Severity of psychotic symptoms was assessed using the Positive and Negative Syndrome Scale (PANSS: [24]). The PANSS is a 30-item clinician-rated scale that assesses three domains of symptomatology: positive symptoms, negative symptoms, and general symptoms. Items are rated such that higher scores are indicative of worse symptomatology.

#### Social and educational/vocational functioning

The Social Functioning Scale (SFS: [25]) was used to measure social functioning among study participants. This 79-item questionnaire assesses six domains of social functioning: social engagement/withdrawal; interpersonal behavior/communication; participation in prosocial activities; participation in recreational activities; independence-competence; and independence-performance. Each domain is scored such that higher scores are indicative of better functioning. A total social functioning score was calculated by summing all SFS subscales into a single variable.

The SFS also provides data with regard to participants’ level of educational/vocational functioning on a 0–10 scale ranging from no perceived capability to work and no active efforts to find a job (0) to participation in full time work/school (10). Consistent with past analyses of educational/vocational functioning among individuals with first-episode psychosis using the SFS [17], these scores were transformed into a dichotomous categorical variable defined as employed/in school (i.e., part-time or greater participation in competitive work/school) versus unemployed/not in school.

#### Cognitive functioning

The MATRICS Consensus Cognitive Battery (MCCB: [26]) was utilized to assess cognitive functioning among study participants. This battery assesses seven domains of cognitive functioning: (i) processing speed; (ii) attention/vigilance; (iii) working memory; (iv) verbal learning; (v) visual learning; (vi) reasoning and problem-solving; and (vii) social cognition. An overall index of cognitive functioning is also computed. Scores are reported as T-scores with higher scores indicative of greater cognitive functioning. The MATRICS offers alternate versions of tests used to assess verbal learning and reasoning and problem-solving to reduce the effect of practice on results from trials in which these measures are administered multiple times. We utilized these alternate forms in the current study and the order in which original and alternate forms were administered was counterbalanced across participants.

#### Substance use

The severity of participants’ substance use was assessed using the Alcohol Use Scale/Drug Use Scale (AUS/DUS: [27]). The AUS/DUS is a clinician-rated scale that assesses use of 12 substances: tobacco, alcohol, marijuana/THC, cocaine, opiates, phencyclidine (PCP), amphetamines, 3,4-methylenedioxy-*N*-methylamphetamine (MDMA), gama-hydroxy butarate (GHB) or flunitrazepam (Rohypnol), huffing of glue or other volatiles, hallucinogens, and other substances of abuse not otherwise specified. Severity of participants’ use is rated on a 5-point scale based on DSM-IV-TR criteria for severity of use: (1) abstinent; (2) use without impairment; (3) abuse; (4) dependence; and (5) dependence with institutionalization. Based on these ratings, participants’ were assigned an overall substance use score using the same 5-point scale. This overall score was calculated as the highest severity ranking earned by a participant across all 12 substance categories.

#### Service utilization and cost-effectiveness

The Service Utilization and Resources Form for Schizophrenia (SURF: [28]) was used to assess participants’ utilization of psychiatric and legal resources. With regard to psychiatric resources, we tracked participants’ use of EPICENTER services as well as non-EPICENTER outpatient mental health services that participants may have chosen to utilize during study participation (e.g., case management, medication management, etc.). Cost outcomes for EPICENTER participants were calculated using cost estimates associated with inpatient hospitalization in Arizona [29] and contact with the legal system [30]. Data with regard to financial support provided by family members was assessed directly in the SURF. Costs associated with unemployment and non-participation in competitive education among young adults were obtained from a recent report from the Corporation for National and Community Service and the White House Council for Community Solutions [31] and included costs associated with lost wages, poor health, and contact with the legal system as well as savings associated with reduced use of government subsidies designed to support participation in higher education. Given that we already obtained estimates of costs associated with contact with the legal system elsewhere, these costs were not included when calculating the total costs associated with unemployment and non-participation in competitive education.

Antipsychotic medication use was assessed using the current medication form—a measure used in past studies of first-episode psychosis [32, 33]. Antipsychotic medications were converted to chlorpromazine equivalents using the conversion values developed by Leucht and colleagues [34] and Woods [35]. Costs for antipsychotic medication used by EPICENTER participants were calculated using 2015 prices for non-generic antipsychotic medication.

Costs for EPICENTER services were calculated using salary and benefits cost data for EPICENTER clinical staff and indirect cost estimates from the hospital where the program is located. Similar to a previous cost-effectiveness evaluation of clinical services for first-episode psychosis [9], costs for non-EPICENTER outpatient mental health services were calculated by multiplying the number of such services by the hourly rate for a mental health counselor as reported in the 2013 National Occupational Employment and Wage Estimates for the United States [36]. Indirect costs for non-EPICENTER outpatient mental health services were calculated using the same indirect rates used for EPICENTER outpatient mental health services. All cost estimates were adjusted to 2015 values to correct for inflation.

### Multi-component intervention

The components of the EPICENTER intervention package were selected following a series of meetings with mental health stakeholders in our local community. Stakeholders included administrators and practitioners in the public and private mental health systems, members of local advocacy groups, and individuals with a mental illness. In these meetings, stakeholders identified access to evidence-based psychosocial interventions as the key treatment need of individuals with first-episode psychosis in our community. Stakeholders verified that the need for psychiatric medication was successfully addressed by existing resources in our community. This information is consistent with existing data on access to evidence-based psychosocial interventions [37–39] and antipsychotic medication [37, 39] published by other research groups in the United States.

Thus, to avoid duplication of existing services, we limited the components of the EPICENTER multi-component treatment package solely to evidence-based psychosocial interventions for psychosis that were otherwise unavailable in our community. All participants interested in receiving antipsychotic medication were able to do so through community providers not affiliated with the study. Selection of components of this treatment package was determined based on the a priori goals to (i) balance the need to address the psychiatric symptomatology and functional deficits (e.g., cognitive functioning deficits) that are part of the core pathology of psychotic disorders with the goal of providing the least time-intensive intervention package for individuals with psychosis [40, 41]; (ii) incorporate interventions that could be applied with fidelity while simultaneously being flexible enough to be responsive to the varying needs of EPICENTER participants [42, 43]; (iii) address the unique gaps in treatment experienced by individuals with psychosis in our catchment area (i.e., limited access to evidence-based psychosocial interventions); and (iv) be delivered effectively given the available staffing resources which ranged from approximately 0.5 to 1.0 full-time positions during the course of the study. The resulting intervention package was comprised of three components: cognitive behavioral therapy, family psychoeducation, and cognitive remediation. Evidence from the study of each respective component suggests that this combined treatment package may address both the symptomatology [44–47] and functional deficits [48–50] common among individuals with psychotic disorders. To increase the flexibility of this intervention package, we selected interventions that could be delivered individually or in group format. Staffing limitations precluded the inclusion of certain evidence-based treatments (e.g., supported employment and education) for which a dedicated full-time staff member is recommended [51, 52].

Upon enrollment in EPICENTER, participants were provided with education with regard to the different elements of the multi-component intervention package. Participants were then allowed to choose which interventions they would complete during their care at EPICENTER.

#### Cognitive Behavioral Therapy (CBT)

CBT is an evidence-based treatment for individuals with psychotic disorders [53] with demonstrated efficacy in both individual [54] and group formats [55]. Of note, though, a recent meta-analysis published after the launch of EPICENTER has called into question the efficacy of this intervention for individuals with psychosis [56]. At EPICENTER we have opted to provide CBT in both an individual and group format. In both formats, we utilize well-established strategies for addressing the positive and negative symptoms that accompany psychotic disorders [57–59] as well as the other sequelae that accompany psychotic disorders, including anxiety [46], insomnia [60], post-traumatic stress disorder [61, 62], substance use [63], and deficits in social and vocational functioning [64–66]. The focus of the intervention is tailored to the specific needs and motivation of the individual with first-episode psychosis such that EPICENTER participants, in collaboration with their therapist, identify the specific therapeutic targets to address in CBT sessions.

#### Family Psychoeducation (FP)

Family psychoeducation is an evidence-based treatment for psychotic disorders [45]. The FP intervention utilized at EPICENTER [67] was based on the protocol developed by McFarlane and colleagues [45] and was modified to address the uniques strengths and challenges of individuals with first-episode psychosis. This intervention involves two modules: (i) joining, (ii) family problem-solving sessions. During the joining module, caregiving relatives meet individually with a clinician for one to three sessions to discuss the patient’s clinical history, the family’s experience and understanding of their relative’s illness, and family members’ concerns and questions with regard to participating in a multifamily group. Following the completion of the joining module, families and their ill relatives have the option to participate in regular family problem-solving sessions (2× per month). During the problem-solving sessions, caregivers and ill relatives identify challenges or problems occurring in their life and evaluate possible solutions to these problems through a structured problem-solving activity. Of note, the family problem-solving sessions, which are the primary component of this FP intervention, are delivered in either a multifamily group or single family format [68] depending on the preference of the family.

#### Metacognitive Remediation (MCR)

Cognitive remediation, which is recognized as a “best practice” in the treatment of psychotic disorders [69, 70], is typically comprised of a series of repeated exercises delivered by a clinician or via a computer that are designed to improve cognitive functioning.

At EPICENTER, participants received metacognitive remediation (MCR: [71, 72])—a form of cognitive remediation shown to improve numerous domains of cognitive functioning among individuals with first-episode psychosis, including processing speed, attention/vigilance, working memory, verbal learning, visual learning, reasoning and problem-solving, and social cognition [71]. MCR involves participation in both computerized cognitive remediation exercises and metacognitive skills development exercises with a clinician. With regard to the former, participants were provided with the computerized CR program PSSCogRehab [73]—a program frequently used in past studies of cognitive remediation in psychotic disorders [74–80]. This program provides participants with training in four areas of cognitive functioning: cognitive foundations (e.g., attention and processing speed), visual-spatial abilities, memory, and problem-solving abilities. Participants initially complete simple tasks in each domain and, once mastered, gradually progress to more difficult tasks. Following each attempt to complete a PSSCogRehab exercise, individuals with first-episode psychosis participate in a “metacognitive discussion” with a clinician designed to promote metacognitive skills development and facilitate transfer of knowledge/skills developed during the MCR session to real-world situations. Of note, not all EPICENTER participants were able to complete MCR during the first 6 months of EPICENTER care due to their participation in another study in which they were randomized not to receive this intervention during the first 6 months of care [81]. In addition, the first 10 EPICENTER participants to receive cognitive remediation did so before the development of MCR, and so completed the same PSSCogRehab tasks but did not participate in metacognitive discussions as described above.

### Statistical analyses

All data were analyzed following the intention to treat principle [82]. Consistent with current statistical guidelines [83, 84], missing data were addressed using multiple imputation [85, 86]. To facilitate the completion of these analyses, change scores were calculated for continuous variables by subtracting baseline assessment values from 6-month assessment values. These values were then analyzed using t-tests. For the investigation of within-subject change in categorical variables, t-values were calculated using effect sizes and standard errors combined using Rubin’s rule [86, 87]. As degrees of freedom calculated using standard metrics for t-tests are overly liberal in the analysis of multiply imputed data, we adjusted the degrees of freedom for our analyses using the formula develop by Barnard and Rubin [88].

Participants who completed both the baseline and 6-month assessments did not differ from those who only completed baseline assessments with regard to symptomatology, cognitive functioning, and most domains of substance use and social functioning. Individuals who did not complete the 6-month assessment reported higher use of marijuana and lower use of tobacco at baseline compared to individuals who completed both the baseline and 6-month assessment. Additionally, individuals who did not complete the 6-month assessment reported better interpersonal communication and competence with regard to the completion of independent tasks of daily living as assessed by the SFS as compared to individuals who completed both the baseline and 6-month assessment.

## Results

### Intervention participation

Rates of participation in EPICENTER interventions are summarized in Table 1. Among all interventions, individual CBT was the most utilized intervention with 61 % of individuals with first-episode psychosis participating in this intervention. Individuals with first-episode psychosis were more likely to participate in individual CBT as compared to group CBT (39 %: *t* = 3.72; *p* < 0.01). Conversely, families were more likely to participate in multifamily (i.e., group) psychoeducation (44 %) as compared to individual family psychoeducation (22 %: *t* = 4.38; *p* < 0.01).

**Table 1:** Rates of participation in EPICENTER interventions

Intervention	Rate of Participation n(%)
Cognitive Behavioral Therapy—Individual	47(61 %)
Cognitive Behavioral Therapy—Group	30(39 %)
Family Psychoeducation—Individual	17(22 %)
Family Psychoeducation—Group	34(44 %)
Metacognitive Remediation	19(25 %)

### Symptomatology

Baseline and 6-month follow-up scores for PANSS are presented in Table 2. Over the first 6 month of care, participants experienced reduction in both positive (*t* = −3.93; *p* < 0.01) and general symptoms (*t* = −4.48; *p* < 0.01). There was no change in severity of negative symptoms from baseline to 6-month assessment (*t* = −1.59; *p* = 0.13).

**Table 2:** Symptomatology, social functioning, cognition, and substance use at baseline and after 6 months of epicenter care

	Baseline	Six Months
PANSS		
1) Positive Symptoms	15.53	13.03*
2) Negative Symptoms	15.07	13.52
3) General Symptoms	30.41	26.29*
SFS		
1) Social Engagement	9.77	9.78
2) Interpersonal Communication	6.60	7.22
3) Prosocial Activities	15.44	18.77
4) Recreation	18.32	20.37
5) Independence-Competence	33.93	36.07*
6) Independence--Performance	22.85	25.47
MCCB		
1) Processing Speed	35.39	41.29*
2) Attention/Vigilance	36.83	38.85
3) Working Memory	40.11	46.59
4) Verbal Learning	40.02	43.98*
5) Visual Learning	38.07	44.89
6) Reasoning and Problem-Solving	40.00	43.85
7) Social Cognition	42.33	49.86
8) Overall Cognitive Composite	32.9	40.16*
AUS/DUS		
1) Overall Substance Use	2.53	1.95*
2) Alcohol	1.98	1.64*
3) Marijuana	1.76	1.39*
4) Tobacco	1.79	1.81

*p < 0.05 as compared to value for baseline assessment

### Social and educational/vocational functioning

Baseline and 6-month follow-up scores for the SFS subscales are presented in Table 2. Overall, there was an increase in total social functioning over the first 6 months of EPICENTER care (total SFS *M* = 108.08 vs. 118.92; *t* = 3.08; *p* = 0.02). With regard to SFS subscales, the independence-competence subscale increased over the first 6 months of EPICENTER care (*t* = 2.73; *p* = 0.03), indicating that participants perceived themselves as more competent in the independent completion of tasks of daily living. There was no statistically significant change in any other SFS subscale from baseline to 6-month assessment.

Participation in competitive employment/education increased over the first 6 months of EPICENTER care. More specifically, the percentage of participants engaged in part-time or greater work/school increased from 38 % at baseline to 49 % after 6 months of EPICENTER care (*t* = 5.98; *p* < 0.01).

### Cognitive functioning

Baseline and 6-month follow-up scores for the MCCB are depicted in Table 2. There was a statistically significant increase in the overall composite cognition score for individuals participating in EPICENTER care (*t* = 3.14; *p* = 0.03). With regard to the individual MCCB subscales, there were statistically significant improvements in verbal learning (*t* = 2.54; *p* = 0.01) and processing speed from baseline to 6-month assessment (*t* = 5.05; *p* < 0.01). There was also a trend suggesting possible improvements in visual learning from baseline to 6-month assessment (*t* = 2.03; *p* = 0.08). Of note, the magnitude of these improvements exceed changes that would be expected due to repeated administration of the MCCB alone (i.e., practice effects [89]).

### Substance use

Baseline and 6-month follow-up scores for the AUS/DUS are presented in Table 2. Participants’ overall substance use declined from baseline to 6-month follow-up (*t* = −5.55; *p* = 0.01). Among our sample, the three most frequently used substances at baseline were alcohol (63 %), tobacco (53 %), and marijuana (48 %). Although participants’ use of alcohol (*t* = −2.34; *p* = 0.03) and marijuana (*t* = −3.16; *p* < 0.01) both declined from baseline to 6-month assessment, there was no change in participants’ use of tobacco during the first 6 months of EPICENTER care (*t* = 0.18; *p* = 0.86).

### Service utilization and cost-effectiveness

Service utilization for EPICENTER participants is summarized in Table 3. Individuals with first-episode psychosis participated in an average of 14.94 EPICENTER-related outpatient mental health visits during the first 6 months of EPICENTER care. The number of episodes of inpatient psychiatric hospitalization (*t* = −3.29; *p* < 0.01), nights of inpatient hospitalization (*t* = −2.54; *p* = 0.01), and contacts with the legal system (*t* = −2.11; *p* < 0.04) were lower in the first 6 months of EPICENTER care as compared to the 6 month period prior to the start of EPICENTER care. Conversely, there was a near significant increase in the number of non-EPICENTER outpatient mental health visits during the first 6 months of EPICENTER care (*t* = 2.18; *p* = 0.07). Although antipsychotic medication dose declined from the baseline to 6-month assessment, this change did not meet criteria for statistical significance (*t* = −0.99; *p* = 0.34). Daily doses of antipsychotic medication (chlorpromazine equivalents) would be considered low (i.e., ≤400 mg) at baseline and after 6 months of EPICENTER care [90].

**Table 3:** Service utilization during 6-month period prior to epicenter care versus during first 6 months of epicenter care

	6-Month Period Prior to EPICENTER Care	First 6 Months of EPICENTER Care
Outpatient Mental Health Visits (Non-EPICENTER)	*M* = 14.59	*M* = 26.13
Outpatient Mental Health Visits (EPICENTER)	N/A	*M* = 14.94
Antipsychotic Medication (chlorpromazine equivalent)	*M* = 331.74 mg	*M* = 288.72 mg
Inpatient Hospitalization (Number of Episodes)	*M* = 0.88	*M* = 0.33*
Inpatient Hospitalizations (Number of Days)	*M* = 13.18	*M* = 4.80*
Contact with the Legal System (Number of Episodes)	*M* = 2.00	*M* = 0.73*

*p < 0.05 as compared to value for 6 month period prior to EPICENTER care

**Fig. 2:**
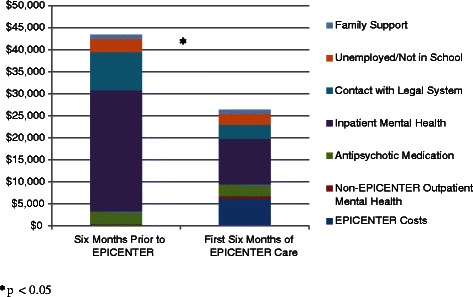
Per Person Service Costs During 6-Month Period Prior to EPICENTER Care versus First 6 Months of EPICENTER Care

Per person cost of services are presented in Fig. 2. The cost of services received by individuals during the 6-month period prior to the start of EPICENTER care (*M* = $43,456) was greater than the cost of services during the first 6 months of EPICENTER care (*M* = $26,355; *t* = −3.00; *p* < 0.01). Care elements contributing to this cost savings included reductions in costs associated with inpatient hospitalizations ($27,480 vs. $10,367; *t* = −3.24; *p* < 0.01) and contact with the legal system ($8,604 vs. $3,169; *t* = −2.10; *p* = 0.04). There was a near significant increase in the costs associated with non-EPICENTER outpatient mental health services during the first 6 months of EPICENTER care ($477 vs. $854; *t* = 2.16; *p* = 0.07). There was no change in costs associated with financial support provided by family members, cost of antipsychotic medications, or costs associated with being unemployed and not in school.

The per person cost of providing EPICENTER care to study participants was $6,136. Dividing the difference of the total costs for the pre-EPICENTER and EPICENTER treatment periods ($17,101) by this value reveals that for every $1 spent on EPICENTER care, $2.79 dollars were saved during the first 6 months of treatment.

## Discussion

The results of the current report highlight the potential clinical effectiveness of a multi-component psychosocial intervention package for first-episode psychosis. On average, individuals participating in EPICENTER care showed improvements in symptomatology, social functioning, educational/vocational functioning, cognitive functioning, and substance use during the first 6 months of treatment. These improvements occurred despite the fact that, on average, participants (i) were already taking antipsychotic medication prior to study enrollment and (ii) did not experience an increase in antipsychotic medication dose during the course of the study. In total, these results add to the growing literature with regard the potency of psychosocial interventions--and multi-component psychosocial intervention packages--provided early in the course of a psychotic disorder [45, 91, 92].

Yet, at the same time, care should be taken to avoid overly-enthusiastic views of the clinical benefits of the EPICENTER intervention package. Certain key outcomes among our participants (i.e., tobacco use and severity of negative symptoms) did not improve over the first 6-months of EPICENTER care. Likewise, although we found improvements in global measures of social and cognitive functioning among individuals with first-episode psychosis, analysis of the subcomponents of these global measures suggests a more conservative interpretation. For example, among the six subcomponents of social functioning used to calculate an overall score for the Social Functioning Scale, only one subscale increased significantly over the first 6 months of EPICENTER care (i.e., independence-competence). Likewise, only two of seven subcomponents used to calculate the overall cognitive functioning score for the MATRICS Consensus Cognitive Battery increased during the first 6 months of EPICENTER care (i.e., verbal learning and processing speed). Moreover, with the exception of the social cognition subscale, performance on the remaining subscales remained 0.5–1.0 standard deviations below the norm for individuals without psychotic disorders (i.e., *T* = 50) following 6 months of EPICENTER care. In total, these data highlight the need for continued investigations of intervention strategies with which to further improve clinical and functional outcomes among individuals with first-episode psychosis.

With regard to service utilization and cost of care, the data are more encouraging. More specifically, the average cost of care during the first 6 months of EPICENTER participation was lower than the average cost during the 6 months prior to joining EPICENTER. These savings occurred despite the additional costs associated with the receipt of EPICENTER care and were driven primarily by reductions in the utilization of inpatient psychiatric services and contacts with the legal system. These savings are especially valuable given the high cost of care of individuals with first-episode psychosis. More specifically, the per person 12-month cost of care among our sample of individuals with first-episode psychosis was $69,810—a cost value noticeably greater than that reported in other cost of care studies that did not limit their sample to individuals early in the course of their psychotic illness [5, 93, 94].

Despite the focus of the current investigation on psychosocial treatments, it is important not to overlook the importance of pharmacological interventions in the treatment of first-episode psychosis. The clinical benefits of such interventions are well documented in the psychosis literature [95]. As noted earlier, most participants were already taking antipsychotic medication prior to study enrollment, and the average per-participant dose of antipsychotic medication did not decline over the course of the study. Given the high rates of medication discontinuation/non-compliance reported in naturalistic studies of individuals with first-episode psychosis [96], the relative stability of medication dose among EPICENTER participants is particularly noteworthy and may have contributed to the positive clinical and cost outcomes among these individuals. Although not formally assessed, it may be that an unintended benefit of participation in EPICENTER care is the facilitation of greater medication adherence among study participants. This hypothesis comports with previous evidence that individuals participating in certain evidence-based psychosocial interventions for psychosis may display very high levels of medication adherence (e.g., multifamily group psychoeducation [68]). Ultimately, future studies are needed to more clearly unpack the association between participation in multi-component psychosocial treatment packages and adherence to pharmacological interventions among individuals with first-episode psychosis.

It is important to note that this study did suffer from a number of limitations—most notably the lack of a study design in which participants were randomly assigned to EPICENTER care versus usual care for first-episode psychosis in our community. With our current study design, it is impossible to rule out the possibility that the improvement in clinical outcomes and cost of care for individuals with first-episode psychosis may simply reflect the natural course of psychotic disorders and are not a result of participation in EPICENTER care. It seems unlikely, though, that this could completely account for our findings given the overwhelming evidence that many of the outcomes investigated in this study (e.g., cognition, social functioning, and educational/vocational functioning) typically worsen or remain stable over the early natural course of psychotic disorders [97–100].

## Conclusions

The results of our study suggest that multi-component psychosocial interventions for first-episode psychosis provided in the US mental health system may be both clinically-beneficial and cost-effective. Although additional research is needed, these findings provide preliminary support for the growing delivery of specialized multi-component interventions for first-episode psychosis within the United States.

## Abbreviations

AUS/DUS: Alcohol Use Scale/Drug Use Scale
CBT: Cognitive Behavioral Therapy
DSM-IV-TR: Siagnostic and Statistical Manual IV-Text Revised
EPICENTER: Early Psychosis Intervention Center
FP: Family Psychoeducation
IQ: Intelligence Quotient
MCCB: MATRICS Consensus Cognitive battery
MCR: Metacognitive Remediation
PANSS: Positive and Negative Syndrome Scale
SFS: Social Functioning Scale
SURF: Service Utilization and Resources form for Schizophrenia
